# Tumor Necrosis Factor-Like Weak Inducer of Apoptosis Accelerates the Progression of Renal Fibrosis in Lupus Nephritis by Activating SMAD and p38 MAPK in TGF-*β*1 Signaling Pathway

**DOI:** 10.1155/2016/8986451

**Published:** 2016-06-05

**Authors:** Zhiqin Liu, Leixi Xue, Zhichun Liu, Jun Huang, Jian Wen, Ji Hu, Lin Bo, Ru Yang

**Affiliations:** ^1^Department of Biological Science & Engineering, Hebei University of Science & Technology, Shijiazhuang 050018, China; ^2^Department of Rheumatology and Immunology, The Second Affiliated Hospital of Soochow University, Suzhou 215000, China

## Abstract

This study aim was to explore the effects of tumor necrosis factor-like weak inducer of apoptosis (TWEAK) in lupus nephritis and its potential underlying mechanisms. MRL/lpr mice were used for* in vivo* experiments and human proximal tubular cells (HK2 cells) were used for* in vitro* experiments. Results showed that MRL/lpr mice treated with vehicle solution or LV-Control shRNA displayed significant proteinuria and severe renal histopathological changes. LV-TWEAK-shRNA treatment reversed these changes and decreased renal expressions of TWEAK, TGF-*β*1, p-p38 MAPK, p-Smad2, COL-1, and *α*-SMA proteins.* In vitro*, hTWEAK treatment upregulated the expressions of TGF-*β*1, p-p38 MAPK, p-SMAD2, *α*-SMA, and COL-1 proteins in HK2 cells and downregulated the expressions of E-cadherin protein, which were reversed by cotreatment with anti-TWEAK mAb or SB431542 treatment. These findings suggest that TWEAK may contribute to chronic renal changes and renal fibrosis by activating TGF-*β*1 signaling pathway, and phosphorylation of Smad2 and p38 MAPK proteins was also involved in this signaling pathway.

## 1. Introduction

The kidney is one of the most commonly affected organs in systemic lupus erythematosus (SLE); about half of the patients with SLE experience lupus nephritis (LN). Although the survival rate for patients with LN has improved to 88% over the last decade, approximately 10%–20% of these patients will develop end-stage renal disease (ESRD) [1]. Fibrotic lesions such as sclerosed glomeruli, interstitial fibrosis, and fibrous vessels are strongly associated with poor renal outcomes in LN. Therefore, the treatment of LN should aim to reduce proteinuria and improve the pathologic features responsible for glomerulosclerosis and tubulointerstitial fibrosis which eventually lead to ESRD, but here we want to stress that repeat biopsy in the clinical treatment is not necessary in most cases. Tubulointerstitial fibrosis is the final common manifestation of various chronic kidney diseases, and progressive accumulation of extracellular matrix (ECM) proteins including collagen 1 (COL-1) in the interstitial area is a key feature [2].

The interaction between the tumor necrosis factor (TNF) superfamily ligands and their receptors is central to the pathogenesis of SLE [3]. Tumor necrosis factor-like weak inducer of apoptosis (TWEAK) is one member of these superfamily ligands, which binds to its receptor, fibroblast growth factor-inducible-14 (Fn14) [4–8]. TWEAK has been shown to play important roles in cell proliferation and differentiation, cell apoptosis, inflammation, and fibrosis through a variety of intracellular signaling pathways, including the TGF-*β* signaling pathway [8–10]. In kidney, TWEAK expression has been reported in mesangial cells, podocytes, endothelial cells, and tubular cells [11]. Increased leukocytes and urinary TWEAK levels are associated with renal disease activity in human LN [12, 13]. In addition to its well-established proinflammatory effects, TWEAK regulates matrix metalloproteinases (MMPs) and tissue inhibitors of MMPS (TIMPs) in myopathies and is associated with the intestinal damage linked to extracellular matrix- (ECM-) related proteins [14–16]. TWEAK-knockout mice were protected from kidney fibrosis in a unilateral ureteral obstruction (UUO) model [10], whereas overexpression of TWEAK gave rise to renal fibrosis in previously normal kidneys. Moreover, anti-Fn14 antibodies were found to reduce residual fibrosis in the acute phase of ischemia reperfusion in mice [17]. These results suggest that TWEAK may contribute to chronic kidney injury and renal fibrosis in LN. In addition, TWEAK-Fn14 interactions also play an important role in the pathogenesis of neuropsychiatric lupus [18, 19]. MRL/lpr Fn14 knockout mice showed significantly reduced neuron degeneration, hippocampal gliosis, and preserved blood brain barrier permeability [18, 19]. So disrupting TWEAK-Fn14 interactions may be an effective approach to the treatment of SLE.

Transforming growth factor-beta (TGF-*β*) signaling, enhanced by proinflammatory cytokines, is a central inducer of renal fibrosis [20]. It has been reported that TGF-*β* can augment TWEAK and Fn14 expression in inflammatory diseases [21]. Conversely, TWEAK enhanced TGF-*β*1 expression in human bronchial epithelial cells and human retinal pigment epithelial cells [22]. Once TGF-*β* is released, the biological activities of TGF-*β* are mediated by activation of the canonical TGF-*β* signaling pathway through binding its receptor 2 (TGF-*β* R2), which then dimerizes with TGF-*β* receptor 1 (TGF-*β* R1) leading to intracellular phosphorylation and activation of the transcription factors Smad2 and Smad3 [23]. Activation of noncanonical TGF-*β* signaling pathway also has been shown to contribute to TGF-*β*-induced renal fibrosis, specifically through the activation of p38 mitogen-activated protein kinase (MAPK) protein [24]. A previous report showed that the single blockade of p38 MAPK after the emergence of established fibrosis is effective in reducing subsequent renal fibrosis in the model of UUO [25]. Among the three isoforms of TGF-*β*, TGF-*β*1 has been identified as the most potent mediator and convergent pathway in renal fibrosis [26].

However, the significance of TWEAK-TGF-*β* signaling pathway in the progression of renal fibrosis of lupus nephritis remains to be determined. We hypothesize that TGF-*β*1 is an important component of TWEAK-induced renal fibrosis in lupus nephritis. To verify our hypothesis, we have studied the role of TWEAK in the activation of TGF-*β*1 through the canonical signaling pathways of TGF-*β* and p38 MAPK* in vivo* and* in vitro*.

## 2. Materials and Methods

### 2.1. shRNA Design and Generation of Lentivirus-Based shRNA

Three pair of shRNAs targeting mice TWEAK gene (Genbank accession number: NM_011614) were designed using the siRNA Target Finder and Design Tool available at http://www.ambion.com/ and were commercially obtained from GeneChem (Shanghai, China). The sequences of these shRNAs were (a) TWEAK-shRNA1 5′-CGGTAACCTACTTTGGACT CTTTCCTCGAGGAAAGAGTCCAAAGTAGGTTATT TTT G-3′ and reverse 5′-AATTCA AAAAAATACTTTGGACTCTTTCCTCGAGGAAAG AGTCCAAAGTAGGTTA-3′, (b) TWEAK-shRNA2 5′-CCGGAATTTACAGTCAT CAGGGCTCTCGAGAGCCCTGATGACTGTAAATTCTTTTTG-3′ and reverse 5′-AATTCAAAAAGAATTTACAGTCATCAGGGCTCTCGAGAGCCCTGATGACTGTAAATTC-3′, and (c) TWEAK shRNA3 5′-CCGGCGAGCTATTGCAGCCCATT ATCTCGA GATAATGGGCTGCAATAGCTCGTTTTTG-3′ and reverse 5′-AATTCAAAAACGAG CTATTGCAGCCCATTATCTCGAGATAATGGGCTGCAATAGCTCG-3′. Each* *of* *these fragments* * was amplified* * and* * ligated* * into* * the* * hU6-MCS-CMV-EGFP (GV115) vector. The nonsilencing shRNA control sequences (LV-Control shRNA) were also designed as a negative control for TWEAK-shRNA (LV-Control shRNA: 5′-CCGGTTC TCCGAACGTGTCACGTTTCAAGAGAACGTGACACGTTCGGAGAATT TTTG-3′ and reverse 5′-AATTCAAAAATTCTCCGAACGTGTCACGTTCTCTTGAAACGTGACACG TTCGGAGAA-3′). 293 T cells were used to detect the silencing efficiency of TWEAK gene by LV-TWEAK-shRNA. TWEAK mRNA and protein levels were detected by Western blotting at 48-hour posttransfection. TWEAK-shRNA1 was the most effective suppressed TWEAK protein expression (results omitted). So we chose TWEAK-shRNA1 as the LV-TWEAK-shRNA for* in vivo* experiments. In this paper, the TWEAK-shRNA is referred to as the TWEAK-shRNA1.

### 2.2. Mice

Thirty-two 13-week-old female MRL/lpr mice were purchased from the Shanghai Slac Laboratory Animal Co., Ltd., and maintained at the Soochow University. Ten MRL/MPJ mice constituted the normal control group (*n* = 10). Animal study protocols were approved by the Animal Care Committee of Soochow University and the experiments were carried out according to the guidelines of the committee. Mice were injected in the tail vein with either 2 × 10^7^ TU LV-TWEAK-shRNA (experimental group, *n* = 11), 2 × 10^7^ TU negative LV-Control shRNA (LV-Control shRNA group, *n* = 11), or phosphate-buffered saline (vehicle control group, *n* = 10) once. All animals were housed in the well-ventilated experimental animal center of Soochow University under specific pathogen-free conditions. Mice were provided ad libitum with food and water and maintained on a natural circadian cycle at 40–70% humidity and a temperature of 20–25°C. All animals were sacrificed by cervical vertebral dislocation four weeks after injection.

### 2.3. Cell Culture

Human proximal tubular cells (HK2 cells) from the Cell Center of Soochow University, Suzhou, China, were grown in a 5% CO_2_ atmosphere at 37°C in Dulbecco's modified Eagle's medium (DMEM) containing 10% fetal bovine serum (FBS) in six-well plastic plates. Once cells reached approximately 80% confluence, they were serum-starved in DMEM containing 0.2% FBS overnight before treatment with or without recombinant human TWEAK (hTWEAK, R&D Systems, Minneapolis, MN, USA) at 100 ng/mL or with TGF-*β*1 at 1 ng/mL (Sigma, St. Louis, MO, USA). In our previous studies, we have found that coculture with a concentration of 10 *μ*g/mL anti-TWEAK mAb could functionally inhibit the role of TWEAK in cultured PBMC, so, in this study, we also cocultured the hTWEAK-stimulated HK2 cells with 10 *μ*g/mL anti-TWEAK monoclonal antibody (mAb; R&D Systems, Inc., USA), or with 10 *μ*M SB431542 (TGF-*β*1 receptor kinase inhibitor, Sigma-Aldrich, Saint Louis, USA) or dimethyl sulfoxide alone. After 24 h, the cells were collected and the expression of TWEAK, p-p38 MAPK, p-Smad2, TGF-*β*1, alpha-smooth muscle actin (*α*-SMA), COL-1, and E-cadherin proteins in HK2 cells were detected by Western blotting.

### 2.4. Histopathology

The kidneys were dissected and fixed in 10% buffered formalin, and paraffin-embedded sections of kidney tissues (3 mm thick) were stained with hematoxylin and eosin (H&E), periodic acid-Schiff (PAS), and Masson's trichrome for histopathological examinations. PAS-positive deposits were each scored from 0 to 4 (0, absent; 1, mild; 2, mild-moderate; 3, moderate; and 4, severe). The presence of inflammatory cell infiltrates determined by H&E staining and the quantification of Aniline Blue-positive areas (indicating fibrotic areas) determined by Masson's trichrome staining were each graded on a scale of 0 to 4 (0, absent; 1, present in <25% of the section; 2, present in 25–50% of the section; 3, present in 50–75% of the section; and 4, present in >75% of the section).

### 2.5. Urine Analysis

To determine renal injury in MRL/lpr mice, urine samples were collected at 17 weeks of age using metabolic cages for 24 h. Urine protein concentrations were measured by a bicinchoninic acid (BCA) protein assay (Thermo Fisher, Waltham, MA, USA).

### 2.6. Western Blotting

The kidneys or cells were lysed with Nonidet P-40 (NP-40) lysis buffer as previously described [27]. Protein concentrations were determined with the BCA Protein Assay Kit (Thermo Fisher Scientific, Waltham, MA, USA). From each sample preparation, 60 *μ*g of total proteins was mixed in Laemmli loading buffer, boiled for 5 min, separated by 10% SDS-PAGE, and then transferred to PVDF blotting membranes (Millipore, MA, USA). Membranes were blocked with Tris-buffered saline-Tween/1% nonfat dry milk and incubated overnight at 4°C with goat antibodies specific for TWEAK (1 : 500, Santa Cruz, CA, USA), rabbit antibodies specific for p-p38 MAPK and p38 MAPK (1 : 500; Santa Cruz, CA, USA), goat antibodies specific for TGF-*β*1 (1 : 250; Santa Cruz, CA, USA), mouse antibodies specific for COL-1, *α*-SMA, and E-cadherin (1 : 500; Abcam, Cambridge, UK), rabbit antibodies specific for p-Smad2 and Smad2 (1 : 500; Abcam, Cambridge, UK), and rabbit polyclonal antibody to *β*-actin (1 : 1000; Sigma Aldrich, Saint Louis, USA). After a final incubation with a 1 : 10,000 dilution of horseradish peroxidase-conjugated secondary antibodies (Zhongshan Biotechnology, Beijing, China) for 2 h at room temperature, the membranes were developed with an ECL detection system (Santa Cruz Biotechnology, CA, USA) and exposed to X-ray film from 30 s to 3 min to visualize chemiluminescent proteins.

Each sample was run in triplicate. The levels of TWEAK, TGF-*β*1, p-p38 MAPK, p-smad2, COL-1, *α*-SMA, and E-cadherin were evaluated by a FluorChem FC2 system (NatureGene Corp., New Jersey, USA). Data are expressed as the ratio of TWEAK, TGF-*β*1, COL-1, *α*-SMA, and E-cadherin band integral optical density (IOD) and *β*-actin IOD, the ratio of p-p38 MAPK IOD and p38 MAPK IOD, or the ratio of p-smad2 IOD and smad2 IOD in the same samples.

### 2.7. Immunohistochemistry Staining of Kidney Sections

Immunohistochemistry was performed on kidney paraffin sections with goat antibodies specific for Fn14 (1 : 300, Santa Cruz, CA, USA), goat antibodies specific for TWEAK (1 : 500, Santa Cruz, CA, USA), mouse antibodies specific for COL-1 and *α*-SMA (1 : 500; Abcam, Cambridge, UK) using the Streptavidin-Biotin Complex (SABC) immunohistochemical technique (SABC kit; Boster Biological Technology Ltd., Wuhan, China). Mean histologic score for each mouse was obtained in a blinded manner in glomerular (20 random glomeruli) and interstitial (20 random fields) sections and each was graded on a scale of 0 to 4 (0, absent; 1, present in <25% of the section; 2, present in 25–50% of the section; 3, present in 50–75% of the section; and 4, present in >75% of the section) [28].

### 2.8. Statistical Analysis

All data were expressed as mean ± standard deviation. A one-way analysis of variance (ANOVA) test was used for comparison of more than two groups. The differences between the groups were assessed with the post hoc Bonferroni test. The datasets were analyzed using the SPSS v 13.0 statistical package. Each experiment was repeated at least 3 times to assess reproducibility. A *p* value of 0.05 was considered as statistically significant.

## 3. Results

### 3.1. LV-TWEAK-shRNA Treatment Alleviated Histopathological Changes in Kidneys of MRL/lpr Mice

**Figure 1:**
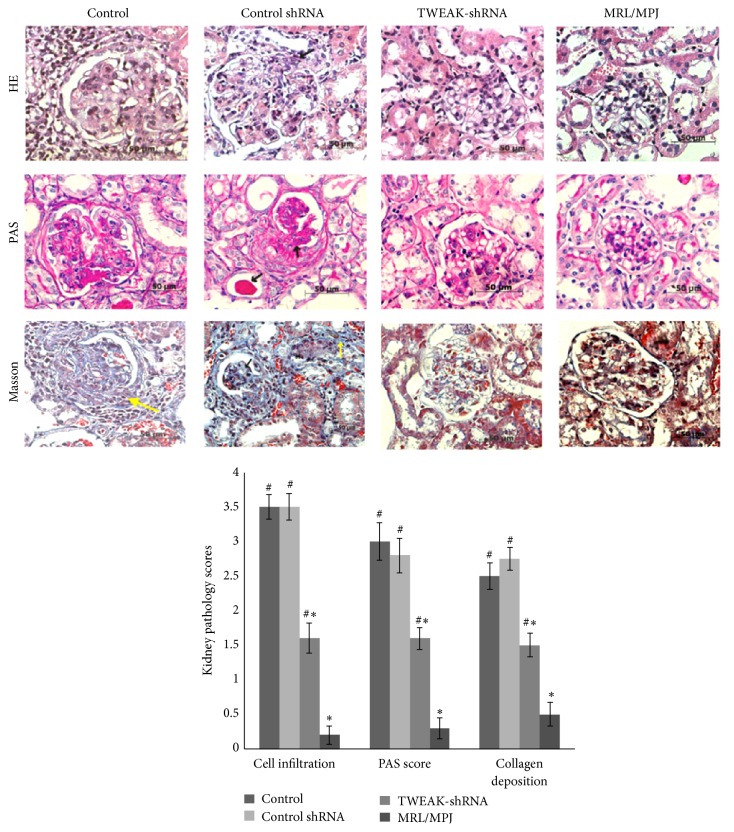
LV-TWEAK-shRNA treatment alleviated histopathological changes in kidneys of MRL/lpr mice. HE representative hematoxylin and eosin- (H&E-) stained sections of formalin-fixed kidneys. PAS representative micrographs of periodic acid-Schiff- (PAS-) stained sections from paraffin-embedded tissues. Masson representative micrographs of Masson-stained sections of paraffin-embedded tissue of the glomerular and interstitial. The kidney pathological scores of cell infiltration and PAS-positive and collagen deposition were shown at the bottom. Compared with those from MRL/MPJ mice, the kidneys from MRL/lpr mice which were treated with PBS or LV-Control shRNA had severe inflammatory infiltrates and histopathology changes, such as a large PAS-positive deposit in the glomeruli (black arrows), segmental overlying cellular crescent sclerosis, crescents, and periglomerular infiltrates, and accumulation of numerous fibroblasts (yellow arrows); damaged kidney tubules and blood vessels were also evident. The kidneys from MRL/MPJ mice showed intact glomerular and tubulointerstitial structures and very few fibroblasts. Control: MRL/lpr mice treated with PBS; Control shRNA: MRL/lpr mice treated with LV-Control shRNA; TWEAK-shRNA: MRL/lpr mice treated with LV-TWEAK-shRNA; and MRL/MPJ: MRL/MPJ mice as normal control. The error bars represent the standard deviation. ^#^ *p* < 0.05 versus MRL/MPJ mice and ^*∗*^ *p* < 0.05 versus control MRL/lpr mice. Scale bar: 50 *μ*m.

**Figure 2:**
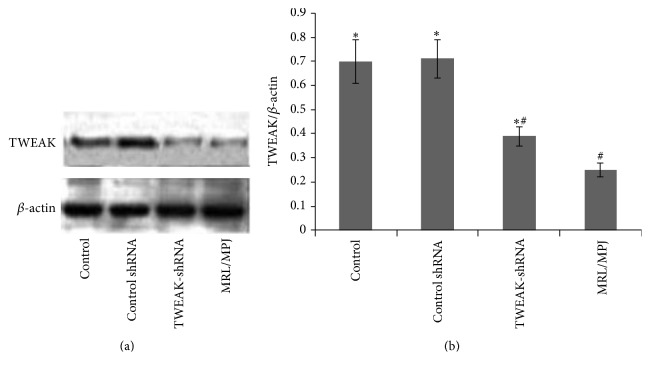
The expression of TWEAK protein in the kidneys. Total lysates of kidneys were collected for Western blotting to measure TWEAK protein expression. The representative experiment was presented (a). The ratios of TWEAK/*β*-actin IOD were shown (b). Control: MRL/lpr mice treated with PBS; Control shRNA: MRL/lpr mice treated with LV-Control shRNA; TWEAK-shRNA: MRL/lpr mice treated with LV-TWEAK-shRNA; and MRL/MPJ: MRL/MPJ mice as normal control. The error bars represent the standard deviation. ^*∗*^ *p* < 0.05 versus MRL/MPJ mice and ^#^ *p* < 0.05 versus control MRL/lpr mice.

Only one mouse in the vehicle control group died during the study. Compared to age-matched MRL/MPJ mice, MRL/lpr mice showed large amounts of lymphocyte invasion, mesangial and glomerular endothelial cell proliferation, glomerular sclerosis, and casts in the tubules, which was accompanied by accumulation of numerous fibroblasts in the renal interstitial area and glomerular area and around the blood vessels (Figure 1). Histological assessment blindly scored by a pathologist revealed moderate to severe glomerular damage and signs of perivascular inflammation in all of the vehicle control and LV-Control shRNA treated mice. These pathological changes were associated with a significant increase in TWEAK protein expression in the kidneys (Figure 2) and elevated proteinuria levels (Table 1). In contrast, the LV-TWEAK-shRNA treated MRL/lpr mice showed a much milder degree of focal glomerular cell proliferation, inflammatory cell infiltration, and fibroblast accumulation in the kidneys. Besides, capillary wall thickening with PAS-positive material deposition was apparently attenuated (Figure 1). TWEAK protein expression in the kidney and proteinuria also decreased significantly (Figure 2 and Table 1).

### 3.2. Distribution of TWEAK and Fn14 Proteins in Kidneys of MRL/lpr Mice

**Figure 3:**
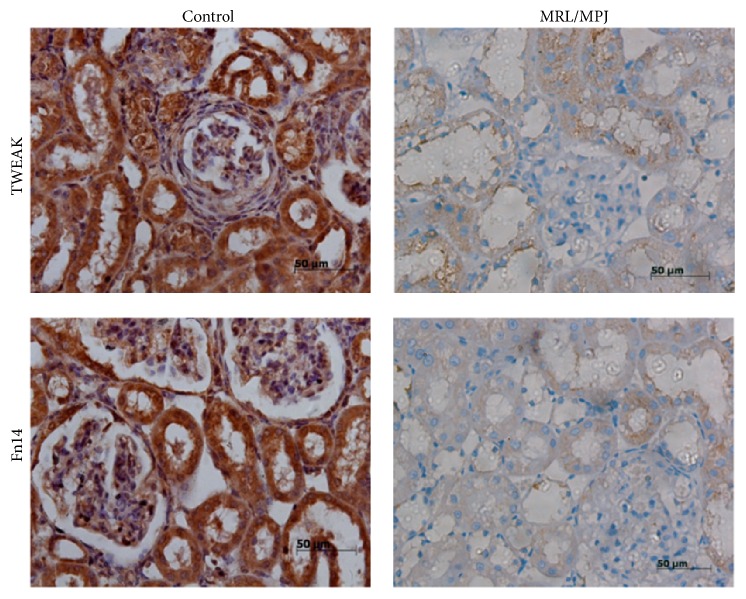
Distribution of TWEAK and Fn14 in kidneys of MRL/Lpr mice. Immunohistochemistry staining of kidneys shows strong expression of TWEAK and Fn14 in the kidneys from MRL/lpr mice, but they were only weakly seen in the kidney of MRL/MPJ mice. Control: MRL/lpr mice treated with PBS and MRL/MPJ: MRL/MPJ mice as normal control. Scale bar: 50 *μ*m.

In this study, the immunohistochemistry results showed that TWEAK and Fn14 proteins were mainly distributed in the glomerular area, tubules, and inflammatory cells of the kidneys in MRL/lpr mice but were only weakly observed in kidneys of MRL/MPJ mice (Figure 3).

### 3.3. Effects of LV-TWEAK-shRNA Treatment on the Expressions of *α*-SMA and COL-1 Proteins in Kidneys of MRL/lpr Mice

It is well known that fibroblast proliferation, altered expression, and overdeposition of extracellular matrix contribute to progressive renal fibrosis. Collagen overproduction, including COL-1, is the major contributor to renal fibrosis [29]. Myofibroblasts in the kidney have also been considered to represent an activated population of resident fibroblasts, which are commonly identified by their expression of *α*-SMA [30]. So we also investigated the role of TWEAK on *α*-SMA and COL-1 expressions in kidneys of MRL/lpr mice.

**Figure 4:**
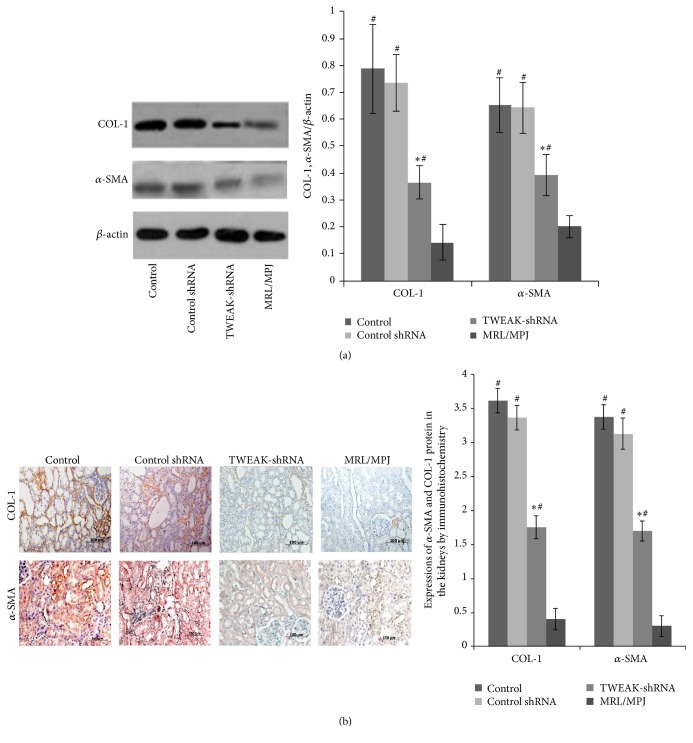
Effects of LV-TWEAK-shRNA treatment on expressions of *α*-SMA and COL-1 proteins in kidneys of MRL/lpr mice. (a) Total lysates of kidneys were collected for Western blotting to measure COL-1 and *α*-SMA protein expressions. The representative experiment was presented (left). The ratios of COL-1/*β*-actin IOD and *α*-SMA/*β*-actin IOD were shown (right). (b) Distribution of *α*-SMA and COL-1 proteins in the kidney was tested by immunohistochemistry. The histologic scores of *α*-SMA and COL-1 proteins were shown at the bottom. Control: MRL/lpr mice treated with PBS; Control shRNA: MRL/lpr mice treated with LV-Control shRNA; TWEAK-shRNA: MRL/lpr mice treated with LV-TWEAK-shRNA; and MRL/MPJ: MRL/MPJ mice as normal control. The error bars represent the standard deviation. ^#^ *p* < 0.01 versus MRL/MPJ mice and ^*∗*^ *p* < 0.01 versus control MRL/lpr mice. Scale bar: 100 *μ*m.

Compared with MRL/MPJ mice, control MRL/lpr mice treated with vehicle showed higher expression levels of COL-1 and *α*-SMA proteins in kidneys by Western blotting and immunohistochemistry; however, LV-TWEAK-shRNA treatment of MRL/lpr mice significantly decreased expression levels of COL-1 and *α*-SMA proteins in kidneys. LV-Control shRNA treatment had no effect on *α*-SMA and COL-1 protein expressions (Figures 4(a) and 4(b)).

### 3.4. Effects of LV-TWEAK-shRNA Treatment on Expressions of TGF-*β*1, p-p38 MAPK, and p-Smad2 Proteins in Kidneys of MRL/lpr Mice

To investigate the effects of TWEAK on TGF-*β* signaling pathway, we determine the expression levels of TGF-*β*1 and its associated downstream signaling pathway proteins (p-p38 MAPK and p-Smad2) in kidneys of MRL/lpr mice after LV-TWEAK-shRNA treatment.

**Figure 5:**
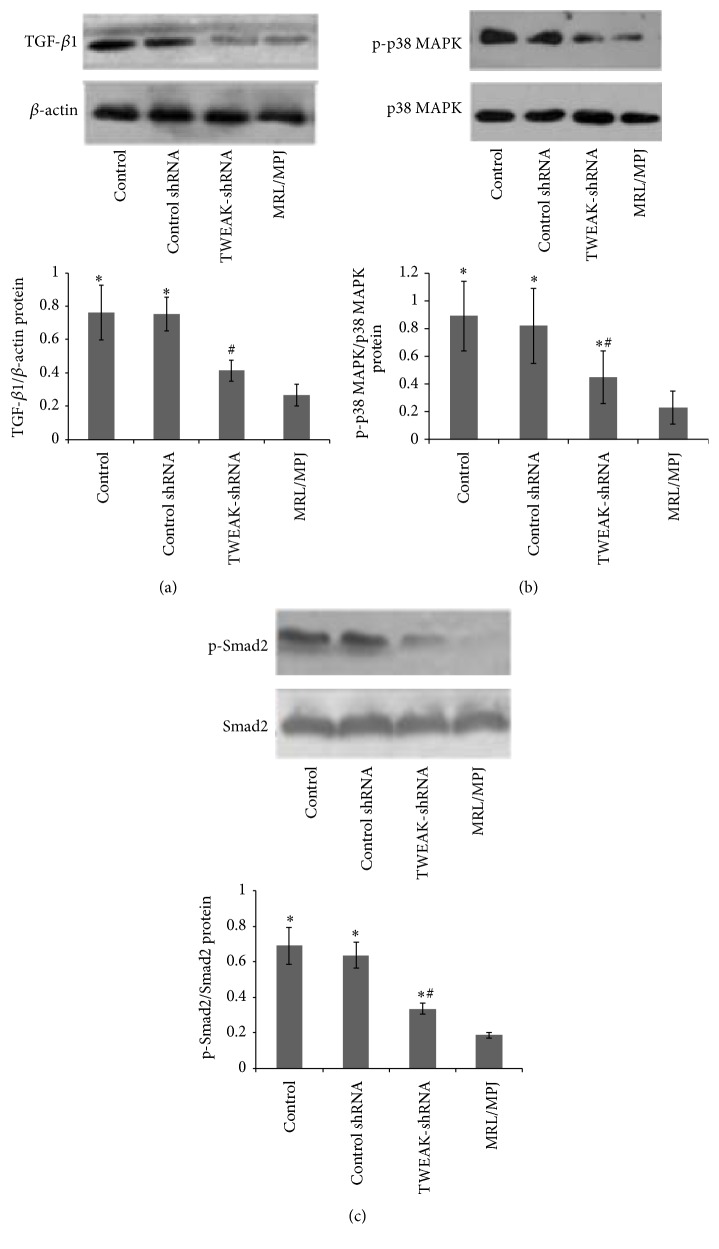
Effects of LV-TWEAK-shRNA treatment on expressions of TGF-*β*1, p-p38 MAPK, and p-Smad2 proteins in kidneys of MRL/lpr mice. Total lysates of kidneys were collected for Western blotting to measure TGF-*β*1 (a), p-p38 MAPK (b), and p-Smad2 (c) protein expressions in MRL/lpr mice. The representative experiments were presented at the top. The ratios of TWEAK/*β*-actin IOD, p-p38 MAPK/p38 MAPK IOD, and p-Smad2/Smad2 IOD were shown at the bottom. Control: MRL/lpr mice treated with PBS; Control shRNA: MRL/lpr mice treated with LV-Control shRNA; TWEAK-shRNA: MRL/lpr mice treated with LV-TWEAK-shRNA; and MRL/MPJ: MRL/MPJ mice as normal control. The error bars represent the standard deviation. ^#^ *p* < 0.01 versus control MRL/lpr mice and ^*∗*^ *p* < 0.01 versus MRL/MPJ mice.

Compared with MRL/MPJ mice, MRL/lpr mice had higher expression level of TGF-*β*1 protein and stronger phosphorylation degree of Smad2 and p38 MAPK proteins in kidneys of control MRL/lpr mice. After LV-TWEAK-shRNA treatment, expression level of TGF-*β*1 and phosphorylation degree of Smad2 and p38 MAPK proteins all decreased in MRL/lpr mice, but LV-Control shRNA treatment had no similar effects (Figure 5).

### 3.5. Effects of SB431542 on hTWEAK Induced Expressions of *α*-SMA, COL-1, and E-Cadherin Proteins in HK2 Cells

E-Cadherin which is localized on the surface of epithelial cells plays an important role in epithelial integrity, and the expression of E-cadherin decreases during the process of renal interstitial fibrosis [31]. So we also show expression of E-cadherin in HK2 cells.

**Figure 6:**
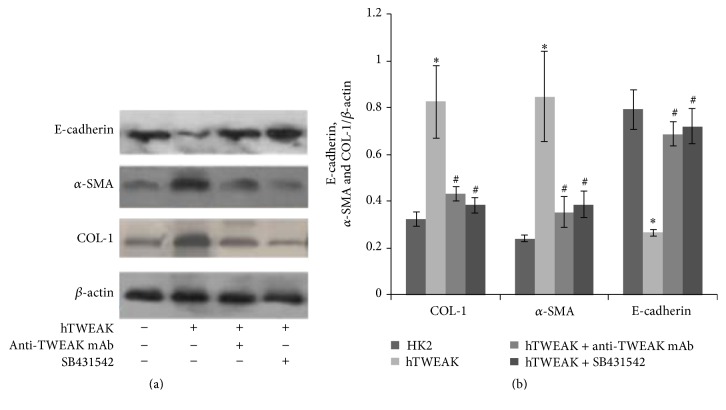
Effects of SB431542 on hTWEAK induced expressions of *α*-SMA, COL-1, and E-cadherin proteins in HK2 cells. Total cell lysates were collected for Western blotting to measure *α*-SMA, COL-1, and E-cadherin protein expressions. The representative experiment was presented in (a). The ratios of *α*-SMA/*β*-actin IOD, COL-1/*β*-actin IOD, and E-cadherin/*β*-actin IOD were shown in (b). HK2: HK2 cells cultured without stimulation; hTWEAK: HK2 cells cultured with hTWEAK; hTWEAK+anti-TWEAK mAb: HK2 cells cocultured with hTWEAK and anti-TWEAK mAb; and hTWEAK+SB431542: HK2 cells cocultured with hTWEAK and SB431542. The error bars represent the standard deviation. Each experiment was repeated at least 3 times. ^*∗*^ *p* < 0.01 versus HK2 cells cultured without stimulation and ^#^ *p* < 0.01 versus HK2 cells cultured with hTWEAK.

After treatment of HK2 cells with hTWEAK for 24 h, the expression levels of *α*-SMA and COL-1 proteins increased significantly, whereas E-cadherin protein expression levels decreased significantly; anti-TWEAK mAb cotreatment diminished the increase of *α*-SMA and COL-1 protein expressions and upregulated E-cadherin protein expression. SB431542, a TGF-*β* receptor 1 inhibitor, showed the same effect as anti-TWEAK mAb and reversed the upregulation of *α*-SMA and COL-1 protein expressions and the downregulation of E-cadherin protein expressions induced by hTWEAK in HK2 cells (Figure 6).

### 3.6. Effects of SB431542 on hTWEAK Induced Expressions of TGF-*β*1, p-Smad2, and p-p38 MAPK Proteins in HK2 Cells

**Figure 7:**
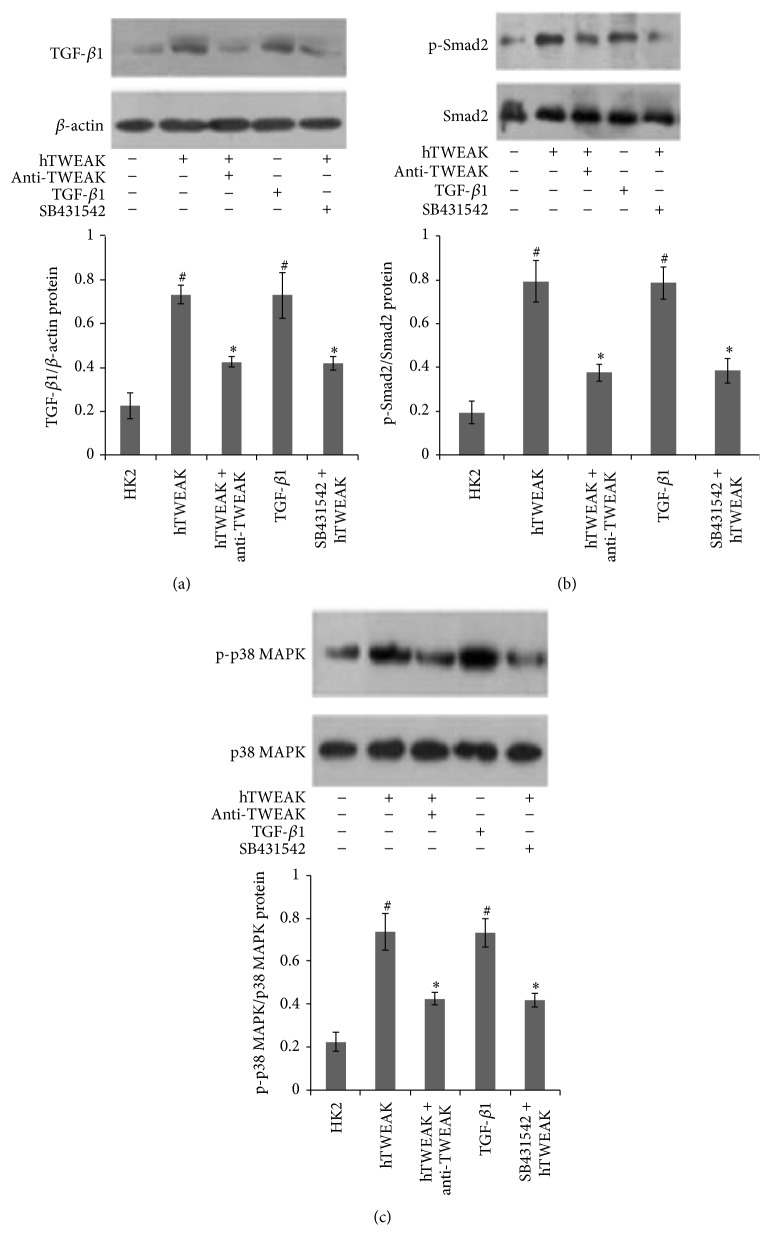
Effects of SB431542 on hTWEAK induced expressions of TGF-*β*1, p-smad2, and p-p38 MAPK proteins in HK2 cells. Total cell lysates were collected for Western blotting to measure TGF-*β*1 (a), p-Smad2 (b), and p-p38 MAPK (c) protein expressions. The representative experiments were presented at the top. The ratios of TWEAK/*β*-actin IOD, p-p38 MAPK/p38 MAPK IOD, and p-Smad2/Smad2 IOD were shown at the bottom. HK2: HK2 cells cultured without stimulation; hTWEAK: HK2 cells cultured with hTWEAK; hTWEAK+anti-TWEAK: HK2 cells cocultured with hTWEAK and anti-TWEAK mAb; TGF-*β*1: HK2 cells cultured with TGF-*β*1; and hTWEAK+SB431542: HK2 cells cultured with hTWEAK and SB431542. The error bars represent the standard deviation. Each experiment was repeated at least 3 times. ^#^ *p* < 0.01 versus HK2 cells cultured without stimulation and ^*∗*^ *p* < 0.01 versus HK2 cells cultured with hTWEAK.

As shown in Figure 7, TGF-*β*1 culture of HK2 cells induced significant phosphorylation of Smad2 and p38 MAPK proteins. hTWEAK treatment had the same effect on phosphorylation of Smad2 and p38 MAPK proteins in HK2 cells and upregulated TGF-*β*1 protein expression. However, anti-TWEAK mAb treatment inhibited upregulation of TGF-*β*1 protein and phosphorylation of Smad2 and p38 MAPK proteins induced by hTWEAK treatment. TGF-*β* receptor 1 inhibitor SB431542 also blocked phosphorylation of Smad2 and p38 MAPK proteins induced by hTWEAK treatment (Figure 7).

## 4. Discussion

MRL/lpr mice are one of the commonly used animal models that show pathologies resembling human SLE, including significant amounts of serum autoantibodies along with immune complex glomerulonephritis and vasculitis [32]. Our experiments demonstrated that MRL/lpr mice at the age of 13 weeks presented with large amounts of proteinuria. At 17 weeks age, the MRL/lpr mice presented with more proteinuria and severe renal pathological changes, such as infiltration of inflammatory cells, collagen deposition, and immune complexes deposition in the capillary wall and basement membrane, which were consistent with the results of other researchers. Moreover, glomerulosclerotic lesions were also demonstrated [33].

TWEAK mRNA level was initially reported to be increased in mouse and human with acute (induced by lipopolysaccharide) or chronic (autoimmune pathologies) inflammatory processes like lupus erythematosus [34]. In contrast to the other TNF ligand-receptor pairs, TWEAK and Fn14 are evidently a monogamous ligand-receptor pair [5]. In this study, we found that TWEAK and Fn14 were weakly expressed in normal condition, while they significantly increased in MRL/lpr mice; the serious pathological changes in the kidneys from MRL/lpr mice were associated with significantly increased renal TWEAK protein. LV-TWEAK-shRNA treatment significantly decreased TWEAK protein expression, accompanied with significantly reductive interstitial and glomerular mesangial matrix deposition, glomerulosclerosis, and interstitial fibrosis in the kidney. Thus we reported that the gene silence of TWEAK alleviated the renal fibrosis of chronic lupus nephritis.

Accumulating evidence suggests that epithelial mesenchymal transition (EMT) is the main process contributing to fibrosis induction [35]. Previous studies have demonstrated that loss of E-cadherin is a critical step in EMT. E-Cadherin is a central component of cell adhesion junctions and is required for epithelial formation and maintaining epithelial homeostasis [31]. The appearance of *α*-SMA in transdifferentiating cells is a hallmark of myofibroblast formation [36]. High renal COL-1 expression is a strong predictor of adverse renal outcome in patients with LN [29]. So we also detected the expressions of *α*-SMA and COL-1 proteins in the lupus mice. We found that *α*-SMA and COL-1 protein levels increased in kidneys of MRL/lpr mice, and the increase was reversed by LV-TWEAK-shRNA treatment. In* in vitro* study, we observed higher expression levels of *α*-SMA and COL-1 proteins and lower expression levels of E-cadherin protein in hTWEAK-stimulated HK2 cells compared with control HK2 cells. It has been reported that overproduction and deposition of ECM components, such as COL-1, progressively replace normal parenchyma and disrupt tissue morphology and function [37]. Considered together, these findings demonstrated that TWEAK could accelerate progression of renal fibrosis in lupus mice through EMT. These findings were consistent with reports from other investigators [38–40]; they have reported that female Fn14-knockout MRL-lpr/lpr mice had significantly low levels of proteinuria and ameliorative kidney proliferative changes in lupus nephritis [41]. Recent studies also showed that TWEAK was an inducer of constitutive TGF-*β*1, PKG, and ERK pathway activation in kidney cells or UUO rats model [10], but the exact mechanism of TWEAK in renal fibrosis in lupus nephritis remains unclear.

As a member of the tumor necrosis factor superfamily ligands, TWEAK has been shown to play an important role in many autoimmune diseases including LN [42]. We found that LV-TWEAK-shRNA treatment downregulated the upregulation of renal TGF-*β*1 protein in MRL/lpr mice; this was also confirmed by* in vitro* study. hTWEAK stimulation resulted in a significant increased TGF-*β*1 protein expression in HK2 cells, and the upregulation was blocked by coculture with anti-TWEAK antibody. Highly expressed *α*-SMA and COL-1 protein and lowly expressed E-cadherin in the HK2 cells by hTWEAK stimulation were also diminished by anti-TWEAK mAb or TGF-*β* receptor 1 inhibitor (SB431542) treatment. As TGF-*β*1 is known to play an important role in renal fibrosis [43, 44], we speculated that TWEAK may participate in renal fibrosis in lupus nephritis through TGF-*β*1 signaling pathway.

In our previous study, we have found that high expression of TWEAK in PBMCs of lupus nephritis patients could activate the p38 MAPK signaling pathway [45]. In addition to induction of leukocyte recruitment to sites of inflammation [45], p38 MAPK is presumed to be involved in cell proliferation induced by TGF-*β* [46, 47] and in TGF-*β*1 induced collagen expression [48]. Previous studies have shown that p38 MAPK is one of the important Smad-independent pathways of TGF-*β* stimulation [49, 50]. Both of the Smad and MAPK pathways have pivotal roles in TGF-*β* signaling regulated renal fibrosis [51]. In these studies, we also observed that the increased protein levels of p-p38 MAPK and p-Smad2 in kidneys of MRL/lpr mice were downregulated by LV-TWEAK-shRNA treatment. We further cultured the HK2 cells with TGF-*β*1; the studies revealed significant phosphorylation of p38 MAPK and Smad2 proteins as in lupus mice. hTWEAK treatment had the same effects as TGF-*β*1 on expressions of p-p38 MAPK and p-Smad2 proteins, and TGF-*β*1 protein in HK2 cells was also upregulated by hTWEAK stimulation. Moreover, both of TGF-*β* receptor 1 inhibitor SB431542 and anti-TWEAK mAb inhibited phosphorylation of Smad2 and p38 MAPK proteins induced by hTWEAK treatment, and anti-TWEAK mAb also decreased TGF-*β*1 protein expression upregulated by hTWEAK treatment. Taken together, we presume that TWEAK mediated positive regulation of TGF-*β* signaling may have a promoting role in kidneys remodeling in lupus nephritis; the phosphorylation of p38 MAPK and Smad2 might be also included in the downstream of TWEAK-TGF-*β* signaling pathway.

In conclusion, TWEAK may contribute to the pathogenesis of kidney remodeling through activating TGF-*β* signaling by inducing the phosphorylation of Smad2 and p38 MAPK proteins in lupus mice. Therefore, blocking TWEAK-TGF-*β*1 signaling pathway may be a promising therapeutic approach in lupus nephritis.

## Figures and Tables

**Table 1:** Proteinuria in MRL/MPJ and MRL/lpr mice (mg/24 h).

	Control	Control shRNA	TWEAK-shRNA	MRL/MPJ
Urine protein (13 weeks)	5.12 ± 0.73	5.34 ± 0.69	5.51 ± 1.03	0.97 ± 0.19
Urine protein (17 weeks)	7.68 ± 1.51^#*∗*^	7.61 ± 1.48^#^	3.16 ± 0.37^#△^	1.25 ± 0.19

Control: MRL/lpr mice treated with PBS; Control shRNA: MRL/lpr mice treated with LV-Control shRNA; TWEAK-shRNA: MRL/lpr mice treated with LV-TWEAK-shRNA; MRL/MPJ: MRL/MPJ mice as normal control. Data are mean ± standard deviation. ^#^ *p* < 0.05 versus MRL/MPJ at the same time point, ^△^ *p* < 0.05 versus control MRL/lpr mice at the same time point, and ^*∗*^ *p* < 0.05 versus control MRL/lpr mice at 13 weeks of age.
